# Highly potent antimicrobial modified peptides derived from the *Acinetobacter baumannii* phage endolysin LysAB2

**DOI:** 10.1038/s41598-017-11832-7

**Published:** 2017-09-13

**Authors:** Shih-Yi Peng, Ren-In You, Meng-Jiun Lai, Nien-Tsung Lin, Li-Kuang Chen, Kai-Chih Chang

**Affiliations:** 1 Department of Laboratory Medicine and Biotechnology, Tzu Chi University, Hualien, Taiwan; 2 Department of Biochemistry, Tzu Chi University, Hualien, Taiwan; 3 Institute of Microbiology, Immunology and Molecular Medicine, Tzu Chi University, Hualien, Taiwan; 4 Department of Laboratory Medicine, Buddhist Tzu Chi General Hospital, Hualien, Taiwan

## Abstract

The increase in the prevalence of multidrug-resistant *Acinetobacter baumannii* (MDRAB) strains is a serious public health concern. Antimicrobial peptides (AMPs) are a possible solution to this problem. In this study, we examined whether AMPs could be derived from phage endolysins. We synthesized four AMPs based on an amphipathic helical region in the C-terminus of endolysin LysAB2 encoded by the *A*. *baumannii* phage ΦAB2. These peptides showed potent antibacterial activity against *A*. *baumannii* (minimum inhibitory concentration, 4–64 μM), including some MDR and colistin-resistant *A*. *baumannii*. Of the four peptides, LysAB2 P3, with modifications that increased its net positive charge and decreased its hydrophobicity, showed high antibacterial activity against *A*. *baumannii* but little haemolytic and no cytotoxic activity against normal eukaryotic cells. The results of electron microscopy experiments and a fluorescein isothiocyanate staining assay indicated that this peptide killed *A*. *baumannii* through membrane permeabilization. Moreover, in a mouse intraperitoneal infection model, at 4 h after the bacterial injection, LysAB2 P3 decreased the bacterial load by 13-fold in ascites and 27-fold in blood. Additionally, LysAB2 P3 rescued sixty percent of mice heavily infected with *A*. *baumannii* from lethal bacteremia. Our results confirmed that bacteriophage endolysins are a promising resource for developing effective AMPs.

## Introduction

The emergence of multidrug-resistant *Acinetobacter baumannii* (MDRAB) strains is a serious public health concern^1, 2^. The increasing prevalence of antibiotic resistance among pathogenic bacteria requires the development of new antimicrobial agents. Antimicrobial peptides (AMPs) hold promise as alternative treatment options to traditional antibiotics.^3^. AMPs are usually small, positively charged and amphipathic with diverse amino acid compositions and length (6–100 amino acids [aa])^4^. Despite their vast diversity, most AMPs kill bacteria in similar ways, consisting of membrane disruption or pore formation that induces an efflux of essential ions and nutrients^5^. Importantly, AMPs are effective against antibiotic-resistant pathogens^6^. Thus far, more than 2,700 natural AMPs with varying sequences have been isolated from various organisms (Antimicrobial Peptide Database [APD], http://aps.unmc.edu/AP)^7^.

Bacteriophages and phage endolysins are suggested to be two of the most promising alternative antimicrobial agents^8^. Their appeal lies in their potency and specificity towards individual bacterial species, typically host bacteria^9^. Exogenous endolysins are highly active against many gram-positive bacteria; however, they are less effective against gram-negative bacteria. This difference in susceptibility is probably due to the barrier function offered by the outer membrane in gram-negative bacteria^10^. However, some endolysins, especially those obtained from phages infecting gram-negative bacteria, can affect bacteria through mechanisms that are completely independent of their enzymatic activity^11–13^. In some of these cases, it was found that amphipathic peptides containing basic amino acids interact with negatively charged membrane elements such as LPS in gram-negative bacteria^9, 11^. In our previous study, we identified endolysin LysAB2 from *A*. *baumannii* phage ΦAB2^14^. LysAB2 showed antimicrobial activity against both gram-positive and gram-negative bacteria. Moreover, LysAB2 treatment enhanced the permeability of the *A*. *baumannii* cytoplasmic membrane. Deletion of the C-terminal region, which contains putative amphipathic helices, significantly reduced the antibacterial activity of LysAB2^14^. Detailed assessment of aa 113–145 of LysAB2 indicated that the sequence had some features similar to those of known AMPs^15, 16^, suggesting that aa 113–145 of LysAB2 had antimicrobial activity. Furthermore, Thandar *et al*. recently showed that the C-terminal region of an *Acinetobacter* phage lysin displayed more lethal activity against *Acinetobacter* than the intact lysin molecule^17^. These clues suggest that *A*. *baumannii* phage endolysins may be a promising source of potent AMPs.

In the present study, we designed and synthesized four AMPs based on the sequence of aa 113–145 of LysAB2 and named these peptides LysAB2 P0-P3. Of the four peptides, LysAB2 P3 showed the highest antibacterial activity against *A*. *baumannii*, but it showed little haemolytic and no cytotoxic activity against normal eukaryotic cells. The effectiveness of the antibacterial activity of LysAB2 P3 in a mouse intraperitoneal infection model was also evaluated.

## Results

### AMP synthesis and *in vitro* antimicrobial assay

The AMPs used in this study are listed in Table 1. The key physicochemical parameters of the peptides were determined using computer programs in the APD^7^. Average hydropathy values and total hydrophobic ratios are summarized in Table 1. To verify whether aa 113–145 of LysAB2 could be used to obtain effective AMPs, we first synthesized a 33-aa peptide called LysAB2 P0. The net charge of the parental peptide LysAB2 P0 was +4.0, and its calculated hydrophobic ratio was 45%. The minimum inhibition concentration (MIC) and the minimum bactericidal concentration (MBC) of LysAB2 P0 against *A*. *baumannii* were both 64 μM (Table 2), indicating that aa 113–145 of LysAB2 (LysAB2 P0 peptide) exerted significant bactericidal effects on *A*. *baumannii*. To enhance the antibacterial activity of LysAB2 P0, we designed and synthesized three other peptides based on LysAB2 P0. The MICs of LysAB2 P1 and P3 for standard *A*. *baumannii* strains were in the range of 4–8 μM, approximately 8–16 times lower than the MIC of LysAB2 P0. The MIC and MBC of LysAB2 P3 against colistin-resistant *A*. *baumannii* were both 8 μM. LysAB2 P1 and P3 showed narrow-spectrum activity only against gram-negative bacteria, especially *A*. *baumannii* (Table 2).

**Table 1:** Amino acid sequences of LysAB2 derivative peptides and prediction of their properties.

AMP	Amino acid sequence (N-terminus to C-terminus)	Length(aa)	MW(kDa)	Average hydropathy value	Total hydrophobic ratio (%)	Total net charge
LysAB2 P0	(LysAB2 aa 113-145)NPEKALEPLIAIQIAIKGMLNGWFTGVGFRRKR	33	3713.463	−0.079	45	+4
LysAB2 P1	**--**EKALE**K**LIAIQ**K**AIKGMLNGWFTGVGFRRKR	31	3560.305	−0.265	45	+6
LysAB2 P2	**--**EKALE**K**LIAIQ**K**AIKGML**A**GWFTGVG**A**RRKR	31	3441.182	−0.126	48	+6
LysAB2 P3	NPEKALE**K**LIAIQ**K**AIKGMLNGWFTGVGFRRKR	33	3765.526	−0.403	42	+6

Amino acid sequences are indicated using the single-letter code; prediction of AMP properties was performed using the APD (http://aps.unmc.edu/AP/)^7^. Different modifications made to LysAB2 are indicated using underscored letters.

**Table 2:** Antimicrobial activity of LysAB2 derivative peptides.

**Antimicrobial activity (μM)**										
**Peptides**		Standard *A*. *baumannii* strains		Clinically isolated colistin-susceptible MDRAB			Clinically isolated colistin-resistant MDRAB		*E*. *coli*	*S*. *aureus*
		ATCC17978	ATCC19606	M3237	M6337	M105656	M2925	M17720	ATCC 25922	ATCC 25923
**LysAB2 P0**	MIC	**64**	**64**	**64**	**ND**	**ND**	**ND**	**ND**	**ND**	**ND**
	MBC	**64**	**64**	**64**	**ND**	**ND**	**ND**	**ND**	**ND**	**ND**
**LysAB2 P1**	MIC	**8**	**8**	**8**	**ND**	**ND**	**ND**	**ND**	**16**	**>128**
	MBC	**8**	**8**	**8**	**ND**	**ND**	**ND**	**ND**	**16**	**>128**
**LysAB2 P2**	MIC	**16**	**16**	**16**	**ND**	**ND**	**ND**	**ND**	**32**	**>128**
	MBC	**16**	**16**	**16**	**ND**	**ND**	**ND**	**ND**	**32**	**>128**
**LysAB2 P3**	MIC	**4**	**8**	**16**	**4**	**8**	**8**	**8**	**32**	**>128**
	MBC	**8**	**8**	**16**	**4**	**8**	**8**	**8**	**32**	**>128**

ND, not done.

### Cytotoxicity assay

**Figure 1:**
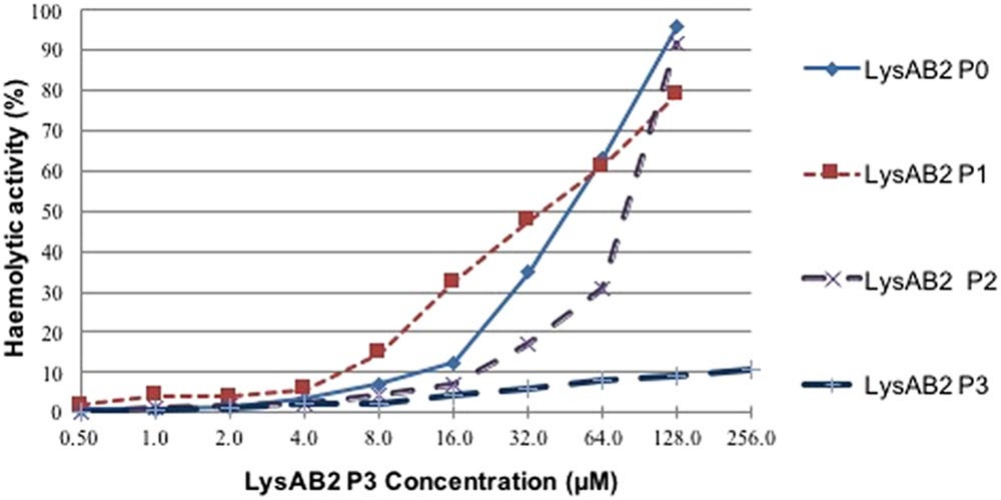
Haemolytic activity of LysAB2 P0, P1, P2, and P3 against human erythrocytes. Haemolysis was determined by measuring the absorbance of the supernatant at 540 nm as an indicator of haemoglobin release. The degree of haemolysis was presented as percentage haemolysis achieved with 0.1% Triton X-100.

**Figure 2:**
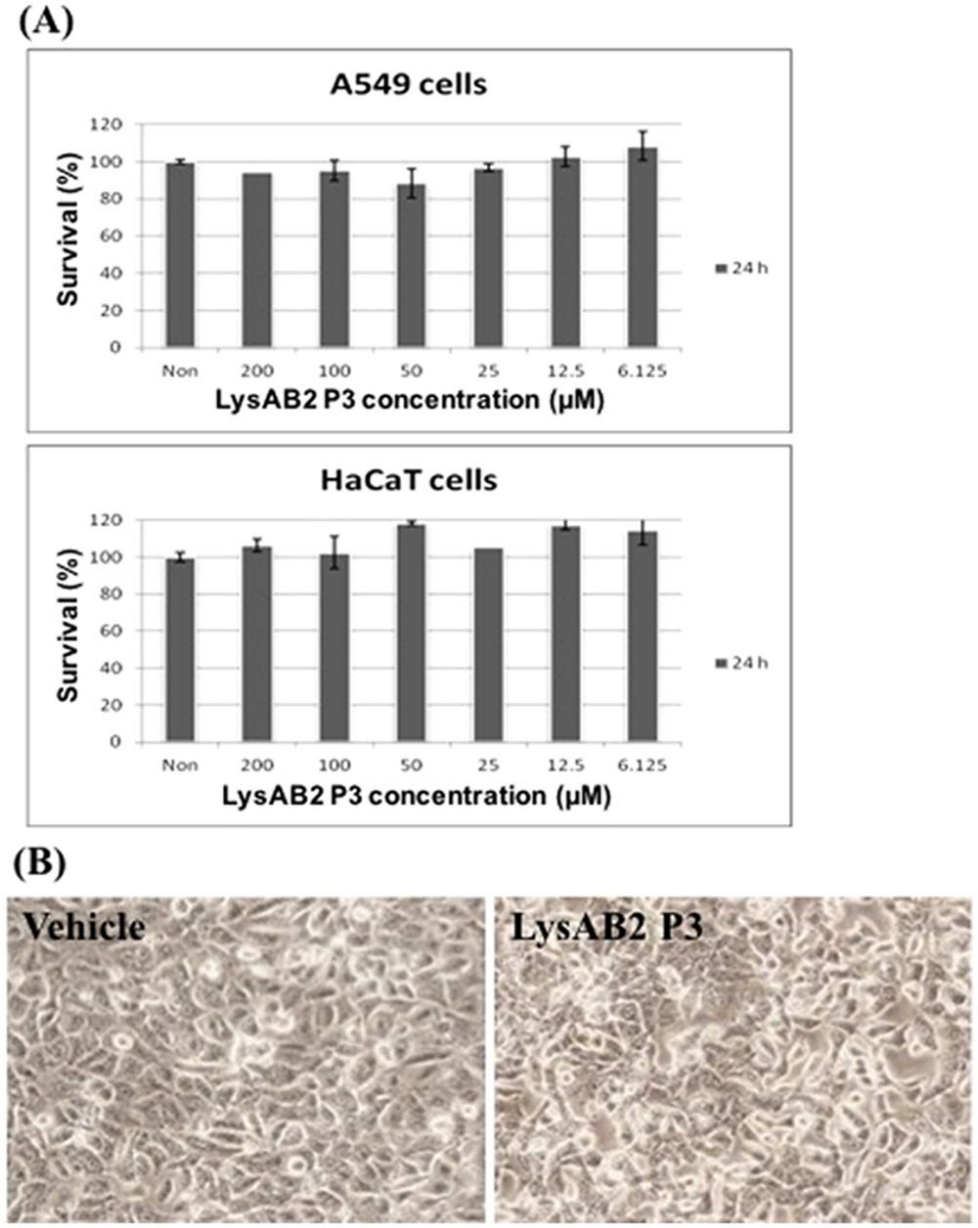
Cytotoxicity assay of LysAB2 P3. (**A**) A549 or HaCaT cells were incubated with various concentrations (0–200 μM) of LysAB2 P3 for 24 h at 37 °C. The effect of LysAB2 P3 on the viability of the cells was determined with an MTT assay. (**B**) No significant alteration was observed in the morphology of HaCaT cells compared with that of untreated cells when examined under an inverted microscope.

The cytotoxicity of LysAB2-derived peptides was determined by measuring their haemolytic activity. Compared with 0.1% Triton X-100, which was used as a 100% haemolytic control, the haemolytic activity of the parental peptide LysAB2 P0 was 63.2% at a concentration of 64 μM (Fig. 1), at which it exerted antimicrobial effects against *A*. *baumannii* strains. In contrast, LysAB2 P3 showed limited haemolytic activity of 1.97% at a concentration of 4 μM, at which it exerted a distinct antimicrobial effect against standard *A*. *baumannii* strains. Increased concentrations of LysAB2 P3 (up to 256 µM) still showed <10% haemolytic activity (Fig. 1), suggesting that LysAB2 P3 had much lower cytotoxicity than the other LysAB2-derived peptides. Next, we performed a 3-(4,5-dimethylthiazol-2-yl)-2,5-diphenyltetrazolium bromide (MTT) assay to determine the effects of different concentrations of LysAB2 P3 on adenocarcinomic human alveolar basal epithelial cells (A549 cells) and aneuploid immortal keratinocyte cells (HaCaT cells). The viability of both cell lines was unaffected after exposure to the peptides for 24 h (Fig. 2A). Moreover, peptide-treated cells showed no significant alteration in their morphology compared with that of untreated cells when examined under an inverted microscope (Fig. 2B).

### Electron microscopy experiments

**Figure 3:**
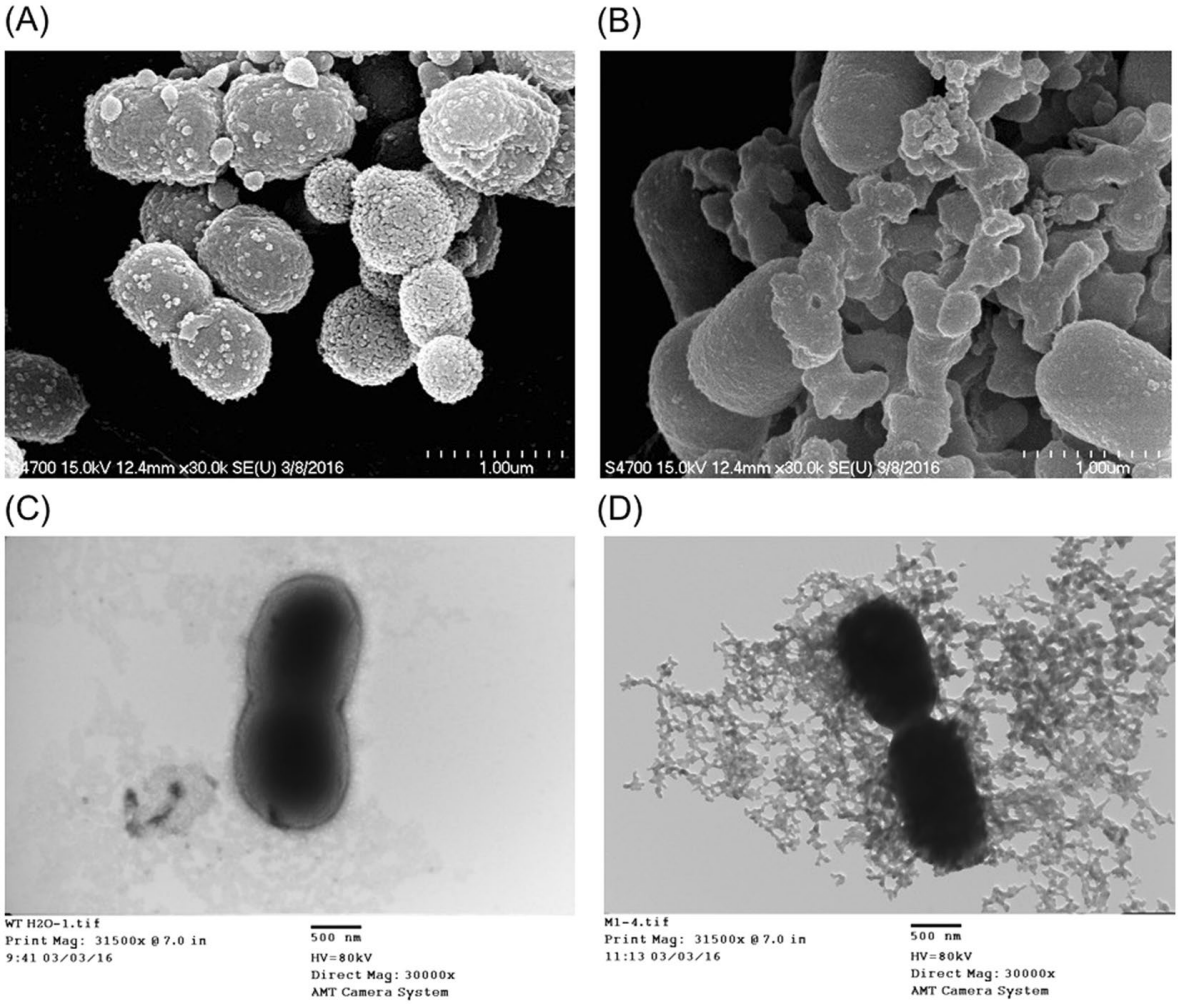
Electron microscopy images of *A*. *baumannii* ATCC 17978. *A*. *baumannii* cells were grown in LB medium and were incubated with PBS (**A**,**C**) or 80 μM LysAB2 P3 (**B**,**D**) for 1 h, then observed by SEM (**A**,**B**) and TEM (**C**,**D**), respectively.

The antimicrobial effect of LysAB2 P3 on the morphology of *A*. *baumannii* strains was determined by performing scanning and transmission electron microscopy (SEM and TEM, respectively). Exposure of *A*. *baumannii* strains to 80 μM LysAB2 P3 for 30 min caused damage to the cell membrane as demonstrated by SEM. Untreated bacteria showed a rough, bright surface, with no apparent cellular debris (Fig. 3A). In contrast, bacteria exposed to LysAB2 P3 showed cell debris and collapse of the cell structure (Fig. 3B). The effect of PBS alone or 80 μM LysAB2 P3 on *A*. *baumannii* was also determined by performing TEM (Fig. 3C,D). The results of TEM showed that *A*. *baumannii* cells treated with 80 μM LysAB2 P3 had membrane damage, with cell envelopes devoid of their cytoplasmic contents and intracellular material found extracellularly (Fig. 3D). These results were similar to those observed by SEM.

### Bacterial cytoplasmic membrane permeation assay

**Figure 4:**
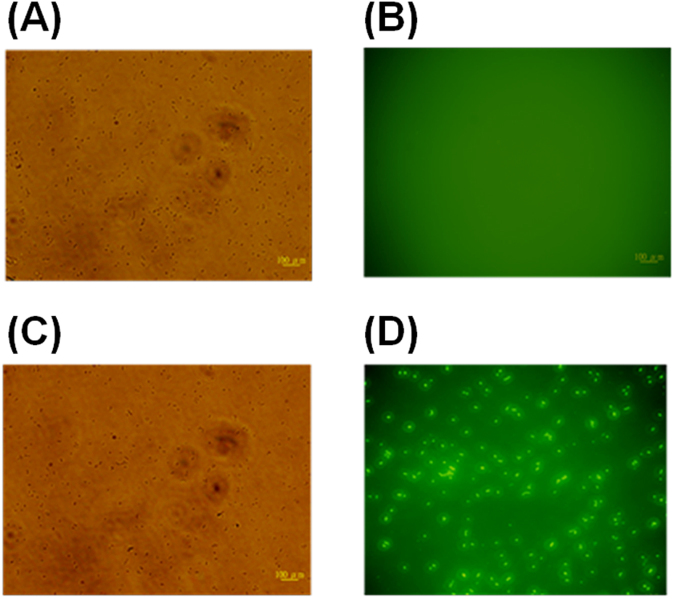
Membrane permeation of *A*. *baumannii* induced by LysAB2 P3 and visualized by FITC staining. To assess the FITC influx induced by LysAB2 P3, we imaged untreated control bacterial cells (**A**,**B**) and LysAB2 P3-treated bacterial cells (**C**,**D**) by bright-field (**A**,**C**) and fluorescence (**B**,**D**) microscopy after FITC staining.

To determine the membrane permeability of LysAB2 P3, we used FITC as a low-molecular-mass (389.4 Da) green fluorescent probe. FITC cannot penetrate the cytoplasmic membrane of intact cells. Incubation of AMP-untreated *A*. *baumannii* with FITC did not produce an appreciable fluorescent signal, as determined by comparing bright-field and fluorescence microscopy images (Fig. 4A,B). In contrast, FITC easily accumulated in bacteria exposed to 8 µM LysAB2 P3 (Fig. 4C and D), suggesting that LysAB2 P3 increased the permeability of the bacterial cytoplasmic membrane.

### Bactericidal activity

**Figure 5:**
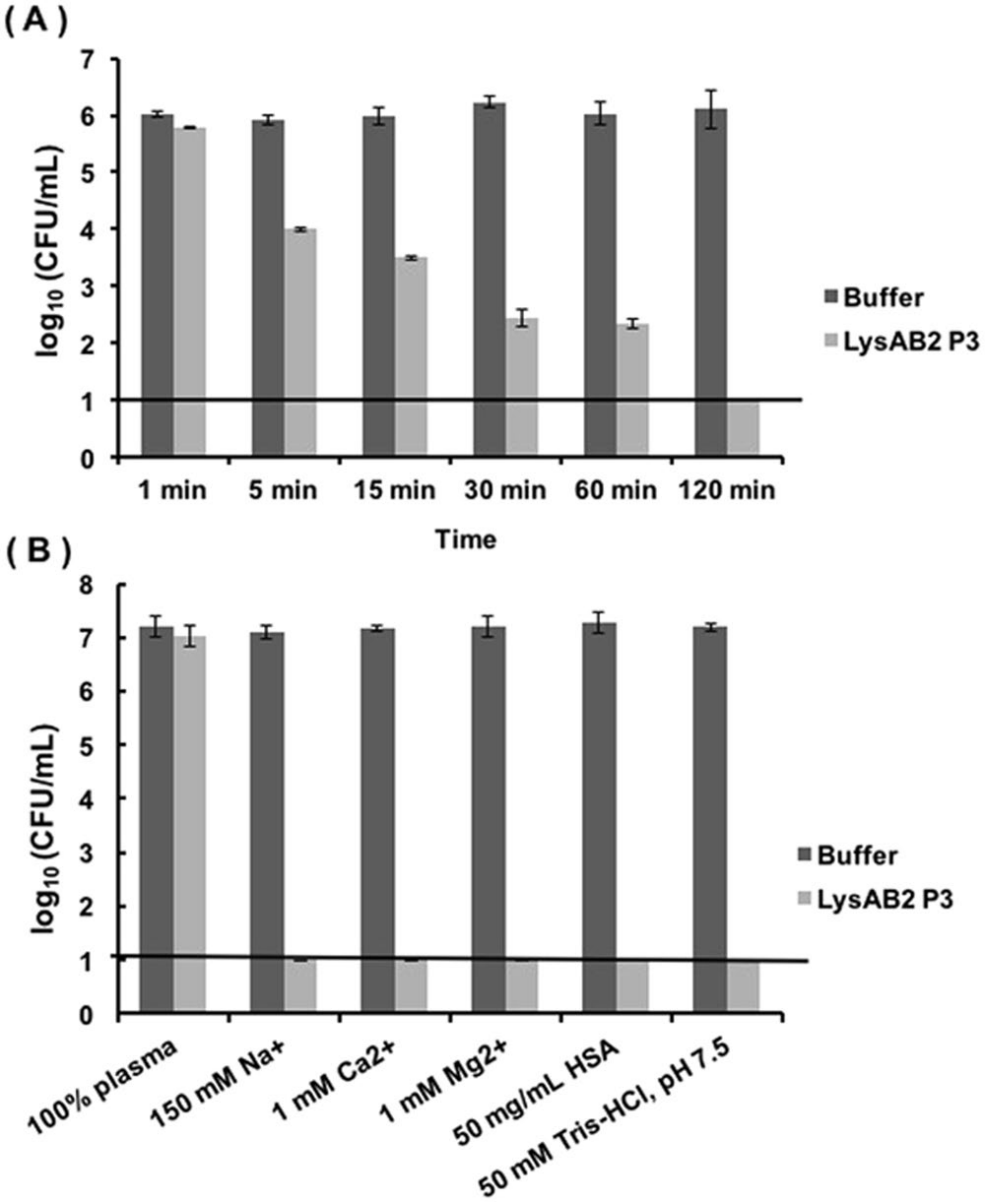
*In vitro* studies of the bactericidal activity of LysAB2 P3 against *A*. *baumannii*. (**A**) Bacterial killing kinetics of LysAB2 P3 against *A*. *baumannii* strain ATCC 17978. (**B**) Bactericidal activity of LysAB2 P3 against *A*. *baumannii* in human plasma and its components. Buffered solution (50 mM Tris-HCl [pH 7.5]) was used as a negative control in both experiments. The error bars show standard deviation, and the black horizontal line marks the limit of detection.

To assess the *A*. *baumannii* kill rate by LysAB2 P3, we performed time-kill assays. An ~2-log decrease in the number of CFU/mL was observed at 5 min, with a continued reduction in bacterial count up to ~5 log at 120 min after LysAB2 P3 addition (Fig. 5A). Since *in vivo* plasma protein binding may modulate the effective binding of AMPs to their targets, we examined the bactericidal activities of LysAB2 P3 in human blood plasma. The result showed that the bactericidal activity of LysAB2 P3 was disrupted and decreased by 68.8% in 100% plasma relative to a reference control in a buffered solution (50 mM Tris-HCl [pH 7.5]). Its activity in the Tris buffer was not disrupted by the addition of monovalent or divalent cations or human serum albumin at physiological concentrations (Fig. 5B).

### Endotoxin release upon LysAB2 P3 peptide treatment

**Figure 6:**
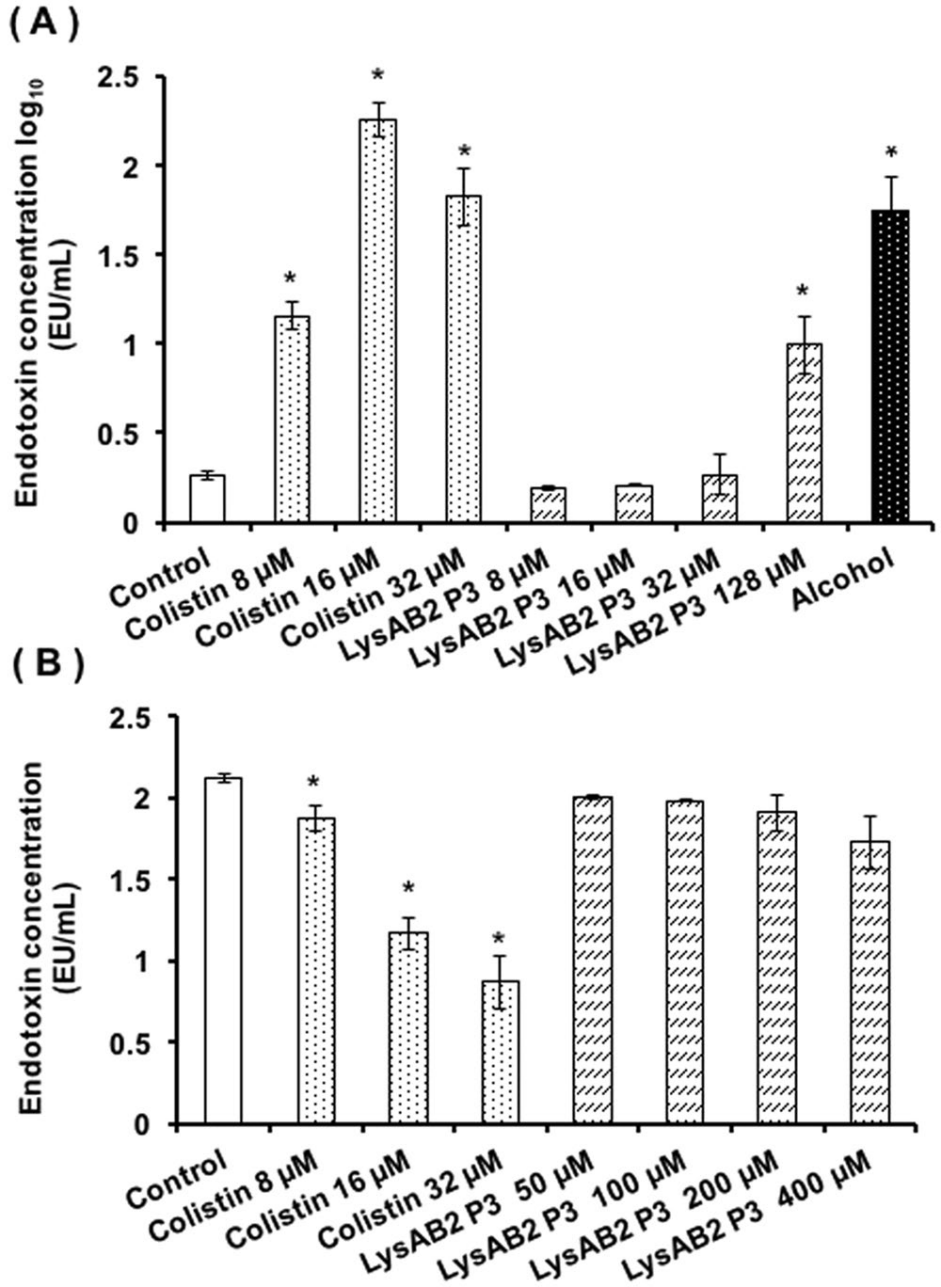
Endotoxin release from *A*. *baumannii* 17978 after exposure to serial concentrations of LysAB2 P3 and colistin. (**A**) The amounts of endotoxin released from *A*. *baumannii* 17978 after incubation with LysAB2 P3 at concentrations ranging from 8 to 128 µM or with colistin at concentrations ranging from 8 to 32 µM. The amount of endotoxin measured in the *A*. *baumannii* group after alcohol treatment was used as the positive control. *p< 0.05. (**B**) Endotoxin neutralization effects of LysAB2 P3 and colistin. LPS from *E*. *coli* O55:H9 was incubated with the indicated concentrations of LysAB2 P3 and colistin, and the remaining biologically active LPS was quantified by an LAL assay. The experiment was conducted in triplicate. *p< 0.05.

After *in vitro* treatment of *A*. *baumannii* 17978 with LysAB2 P3 and colistin, the endotoxin release was measured with a *Limulus* amebocyte lysate (LAL) assay. Statistically significant release of endotoxin from *A*. *baumannii* 17978 was detected only at a high concentration of LysAB2 P3 (128 μM, which is 32 times its MIC), although the endotoxin release level was comparable to that of the cells being treated with 8 μM of colistin (Fig. 6A). No tested concentration of LysAB2 P3 showed statistically significant potency in neutralizing endotoxin from *Escherichia coli* (*E*. *coli*) (Fig. 6B).

### Animal studies

**Figure 7:**
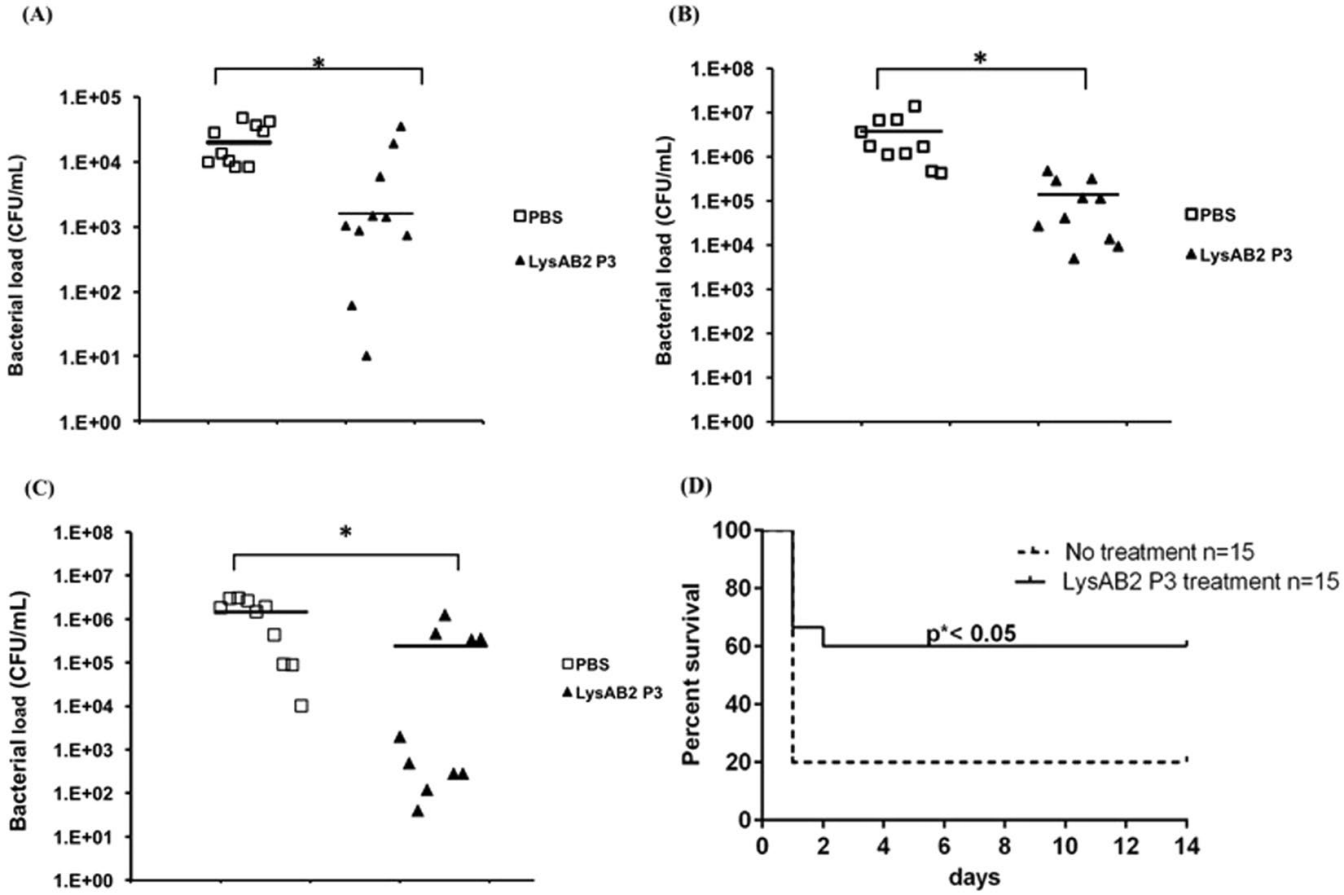
*In vivo* studies of the protective activity of LysAB2 P3 against *A*. *baumannii*. Quantitative comparison of bacterial load in ascites (**A**) and blood (**B** and **C**) samples of mice treated with PBS (open square) versus those of mice treated with LysAB2 P3 (solid triangle) at 1 h (**B**) and 3 h (**C**) after bacterial inoculation. The line indicates mean bacterial load; *p < 0.05 for PBS versus LysAB2 P3. (**D**) The survival of mice treated with a single dose of LysAB2 P3 or PBS buffer i.p. after *A*. *baumannii* infection. The survival of the mice was monitored for 14 days. *p < 0.05 for PBS versus LysAB2 P3.

To investigate the ability of LysAB2 P3 to reduce *A*. *baumannii* load *in vivo*, we inoculated each BALB/c mouse i.p. with 2×10^7^ CFU of *A*. *baumannii* ATCC 17978, followed by i.p. treatment with a single dose of PBS (10 mL/kg) (n = 10) or LysAB2 P3 (100 µM/mouse, 3.7 mg/kg) (n = 10) at 1 h after the bacterial injection. At 4 h after the bacterial injection, the bacterial load in the ascites of control mice was approximately 20,000 CFU/mL (Fig. 7A). Administration of LysAB2 P3 significantly reduced the bacterial load (~1,500 CFU/mL) in ascites by 13-fold compared with that in the ascites of PBS control mice (P <0.05). The *in vivo* antimicrobial activity of LysAB2 P3 was also determined using two experimental conditions in a mouse model of *A*. *baumannii*-induced bacteremia. The mice were i.p. inoculated with *A*. *baumannii* ATCC 17978 (5 × 10^8^ CFU). In the first experiment, the inoculation was followed by intraperitoneal treatment of the mice with 32 µM/mouse (1.2 mg/kg) of LysAB2 P3 (n = 10) after 1 h. In the second experiment, the mice were treated i.p. with 200 μM/mouse (7.5 mg/kg) of LysAB2 P3 at 3 h after the bacterial inoculation. The treatments reduced the bacterial load in mice with bacteremia by 27-fold (Fig. 7B) and 6-fold (Fig. 7C), respectively, compared with those in PBS (10 mL/kg) control mice. Since lethal bacteremia is a common outcome of *A*. *baumannii* infections, we investigated the ability of LysAB2 P3 to work systemically and rescue mice from this type of infection. Each mouse received 5×10^8^ CFU of *A*. *baumannii* i.p. and was treated 3 h later with a single dose of 200 μM/200 μL LysAB2 P3 or buffer by the same route. While most (80%) buffer-treated mice died within 1 day, the LysAB2 P3-treated mice had a significantly higher rate of survival, with 60% being rescued from this highly lethal dose of *A*. *baumannii* (Fig. 7D). These results collectively indicate that LysAB2 P3 has good antimicrobial activity *in vivo*.

## Discussion

The emergence of MDRAB is a serious public health concern^1, 2^. In the past few decades, polymyxins have been increasingly used in clinical settings to treat multidrug-resistant bacteria^1^. Polymyxins exert their bactericidal activity by binding to the bacterial membrane to increase membrane permeability. This disruption results in leakage of intracellular components. In addition to this antimicrobial property, polymyxins can bind to and inactivate endotoxin. Thus, the application of polymyxins in therapeutic strategies to treat sepsis has been investigated^18^. However, polymyxin-resistant bacteria have emerged recently, and studies have shown the adaptive resistance of bacteria to polymyxins^19, 20^. There is an urgent need for more effective treatments to overcome drug resistance in bacteria. Bacteriophages and phage endolysins have been proposed as two of the most promising alternative antimicrobials^8^. We previously evaluated endolysins derived from *A*. *baumannii* bacteriophages for eliminating MDRAB^13, 14^. Lood *et al*. recently also showed that a *A*. *baumannii* phage endolysin was able to successfully control MDRAB infection in a mouse bacteremia model^9^. These endolysins are suggested to exert bactericidal effects by permeating the outer bacterial membrane using their highly positively charged C-termini^9, 13, 14^. Computer programs in the APD^7^ suggested that aa 113–145 of LysAB2 could interact with bacterial membranes and be used as AMPs. In the present study, we designed and synthesized four AMPs (LysAB2 P0-P3) from the C-terminal amphipathic helical region of LysAB2.

The original AMP LysAB2 P0 had a total net charge of +4, a total hydrophobic ratio of 45%, and average hydropathy of −0.079. Its amino acid sequence was predicted to form an alpha helix with at least 8 residues on the same hydrophobic surface. (Calculation was performed using APD3: Antimicrobial Peptide Calculator and Predictor^7^) (Table 1). The MIC and MBC of the synthetic LysAB2 P0 were both 64 µM for all the *A*. *baumannii* strains tested (Table 2), indicating that aa 113–145 of LysAB2 could be used to design AMPs. However, 64 μM LysAB2 P0 had a haemolytic activity of 63.2%. The highest haemolytic activity of almost 100% was observed for 128 μM LysAB2 P0 (Fig. 1). To improve the antibacterial activity and reduce its haemolytic activity but avoid a drastic change in the structure, we modified LysAB2 P0 progressively based on the following principles. (1) Retain the residues predicted to form an alpha helix with at least 8 residues on the same hydrophobic surface in LysAB2 P0. (2) Retain the frequently used amino acids (A, C, G, K, L), and substitute the rarely used amino acids revealed in known antimicrobial peptides^7, 21^. (3) Retain the residues that maintain the original net charge of LysAB2 P0 (E: 2; K: 3; R: 3). Under these principles, the remaining residues that were considered for replacement in LysAB2 P0 were P8, Q13, I14, N21, F24, T25, V27, and F29.

First, we increased the net charge of LysAB2 P0 but retained its original hydrophobic ratio. LysAB2 P1 lacked the first two aa of the N-terminus and was derived from LysAB2 P0 by substituting of K for P8 and I14, as K is a positively charged amino acid frequently used in AMPs. LysAB2 P1 was predicted to gain two more positive charges and maintain its hydrophobic ratio compared with LysAB2 P0. The synthetic LysAB2 P1 showed approximately eight times the antimicrobial activity of LysAB2 P0 (Table 2). Although LysAB2 P1 had lower haemolytic activity than LysAB2 P0, its cytotoxicity limited its clinical usefulness (Fig. 1). We next modified LysAB2 P1 by substituting A for N21 and F24, as A is a hydrophobic molecule frequently occurring in AMPs. The resulting peptide is termed LysAB2 P2 (Table 2). This modification not only increased the hydrophobic ratio of LysAB2 P2 but also placed at least 11 residues on the same hydrophobic surface. The haemolytic activity of LysAB2 P2 was relatively lower than that of LysAB2 P1; however, the modification also slightly decreased its bactericidal activity. The results of the peptides derived so far showed that the increased net charge and hydrophobic ratio improved the bactericidal activity but did not effectively decrease the haemolytic activity. Therefore, we next tried to reduce the hydrophobic ratio of LysAB2 P2 but maintain its net charge by replacing P8 and I14 of LysAB2 P0 with K, yielding LysAB2 P3. LysAB2 P3 had a total net charge of +6, a total hydrophobic ratio of 42%, and an average hydropathy value of −0.403 (Table 1). Notably, LysAB2 P3 was 16 times more active than LysAB2 P0 (Table 2) and had little haemolytic activity against normal erythrocytes (Fig. 1). Therefore, we speculated that the additional positive charge or lysine residues were important for the antimicrobial activity of LysAB2 derivative peptides and that a decreased partial hydrophobic ratio or average hydropathy value reduced the cytotoxicity of these peptides.

We used LysAB2 P3 in all subsequent experiments because it was the most potent peptide and had little haemolytic activity. The MBC values of LysAB2 P3 against both *A*. *baumannii* ATCC 17978 and colistin-resistant *A*. *baumannii* were the same (8 μM, Table 2). We also examined the *in vitro* bactericidal spectra of LysAB2 P3. Among the bacteria tested, *A*. *baumannii* strains were consistently the most sensitive to LysAB2 P3 (average MBC, 8 μM). *E*. *coli* were moderately sensitive to LysAB2 P3, with an MBC that was 4 times higher than that for *A*. *baumannii* strains. However, *S*. *aureus* were resistant to LysAB2 P3 under the same experimental conditions (MBC,>128 μM; Table 2). Therefore, we speculated that LysAB2 P3 had a narrow bactericidal spectrum and was only active against gram-negative bacteria, especially *A*. *baumannii*. The antibiotics polymyxin B and polymyxin E (colistin) are cationic AMPs derived from the soil bacterium *Bacillus polymyxa*. They have good bactericidal effects against gram-negative bacteria but little or no effect on gram-positive bacteria. Polymyxins show high affinity towards LPS and strong interaction with the negatively charged membranes that gram-negative bacteria usually have^18, 22^. This information suggests that the observed bactericidal spectrum of LysAB2 P3 may similarly be due to its different binding affinity and specificity towards the membrane components of different bacteria.

A bacterial cytoplasmic membrane permeation assay was performed to elucidate the antimicrobial action of LysAB2 P3 against *A*. *baumannii*. The results of this assay showed that LysAB2 P3 increased the permeability of the bacterial cytoplasmic membrane. The results of electron microscopy also showed that LysAB2 P3-treated *A*. *baumannii* exhibited cell membrane damage (Fig. 3A and B). This may be because, similar to most AMPs, LysAB2 P3 directly affects microbes by disrupting their membranes^5^. After demonstrating that LysAB2 P3 did not exert any significant cytotoxic effect on human cells, we examined its *in vivo* effect on the bacterial load in infected mice. At 4 h after the bacterial injection, LysAB2 P3 significantly reduced the bacterial load in ascites by 13-fold and in blood by 27-fold compared with PBS-treated mice (Fig. 7A and B). These results indicate that LysAB2 P3 has significant antimicrobial activity *in vivo*. However, the *in vivo* antimicrobial activity of LysAB2 P3 was much weaker than its *in vitro* bactericidal activity, suggesting that the bactericidal or membrane-penetrating activity of LysAB2 P3 was affected by plasma or bodily fluids. *In vivo* plasma protein binding may modulate the antimicrobial activity of AMPs. We first tested the bactericidal activity of LysAB2 P3 in the presence of plasma and its components *in vitro*. The results showed that LysAB2 P3 was disrupted and decreased in activity by 68.8% in 100% plasma (Fig. 5B). However, its bactericidal activity in the Tris buffer was not influenced by the addition of monovalent or divalent cations or human serum albumin at physiological concentrations. In the mouse bacteremia experiments, 3 h after bacteria injection, even though the mice were treated with the higher concentration (200 μM/mice) of LysAB2 P3 via the intraperitoneal route, the ability to reduce the bacterial load in blood was lower than in the 1 h post-infection experiment. However, the results still showed a 6-fold reduction in blood bacterial counts relative to the PBS control. Moreover, encouragingly, while the *in vivo* bacteremia model with i.p. administered LysAB2 P3 was conducted using the same conditions (waiting 3 h after bacteria inoculation before intraperitoneal treatment of the peptides), it resulted in 60% 14-day survival rate, which suggests that LysAB2 P3 was still active in the peritoneal cavity.

The results of our previous studies suggest that the highly cationic C-terminal region of LysAB2 permeabilizes the outer membrane of bacteria^14^. Recently, Lood *et al*. also identified a similar highly positively charged region in the *A*. *baumannii* phage lysin PlyF307^9^ and termed it P307^17^. Although the sequence of LysAB2 P3 is not highly similar to that of P307^17^, these two peptides showed similar bactericidal activity and mechanisms of action. Moreover, both peptides were most effective against *A*. *baumannii* strains, and their bactericidal activity was partially affected by bodily fluids.

In addition to bactericidal activity, some AMPs have been found to have anti-endotoxin abilities whereby they can prevent the release of inflammation-inducing toxins, such as LPS from gram-negative and lipoproteins (lipoteichoic acid and peptidoglycan) from gram-positive bacteria^22, 23^. In this report, the statistically significant release of endotoxin was detected when *A*. *baumannii* was treated with a high concentration (128 µM; 32 times its MIC) of LysAB2 P3 (Fig. 6A). However, no significant anti-endotoxin activity of LysAB2 P3 was detected at the concentrations tested (50, 100, 200 and 400 µM; Fig. 6B). A previous report suggested that the different molecular geometry of LPS on the bacterial surface and at release could affect the anti-endotoxin effect of AMPs. The AMPs with efficient antibacterial activity may not have the same ability to neutralize endotoxin^23^. This may explain the observation that LysAB2 P3 has a strong antibacterial effect against *A*. *baumannii* but has low endotoxin-neutralizing activity. Careful dose monitoring will be needed if LysAB2 P3 is considered for clinical application.

Although rules and models have been proposed for the de novo design of AMPs, several effective AMPs exist in naturally occurring proteins, such as bacteriophage endolysins, and these peptides should be explored. In the present study, we showed that AMPs can be derived from the amphipathic helical region in the C-terminus of the endolysin LysAB2. Of the four peptides, LysAB2 P3, with modifications that increased the net positive charge and decreased the hydrophobicity, showed high antibacterial activity against *A*. *baumannii*, but it had little haemolytic and no cytotoxic activity against normal eukaryotic cells. The results of the present study confirmed that the amphipathic helical region in the C-terminus of *A*. *baumannii* phage-encoded endolysins has the potential to be used for developing novel AMPs.

## Materials and Methods

### Bacterial strains and growth conditions

Antimicrobial activities of LysAB2 derivative peptides were examined using several bacterial strains from the American Type Culture Collection (ATCC), including *A*. *baumannii* strains ATCC 17978 and ATCC 19606, *E*. *coli* strain ATCC 25922, and *Staphylococcus aureus* strain ATCC 25923. Clinical isolates of MDRAB were collected from Buddhist Tzu Chi General Hospital, Taiwan. Susceptibility to colistin was determined using the broth dilution method, in accordance with the guidelines of the Clinical and Laboratory Standards Institute^24^. Bacteria were cultivated in Luria-Bertani (LB) broth or LB agar (Difco Laboratories, Detroit, MI) at 37 °C.

### Analysis and synthesis of AMPs

The AMPs used in this study are listed in Table 1. The properties of these AMPs were predicted using computer programs in the APD^7^. The peptides were synthesized by solid-phase peptide synthesis and were purified by reverse-phase high-performance liquid chromatography to >85% purity using a MISSION BIOTECH system (Taipei, Taiwan). The synthetic peptides obtained were dissolved in ddH_2_O for use in subsequent experiments.

### Antimicrobial activity

The MIC of each designed peptide was determined in three independent experiments using a standard microdilution method in 96-well microtiter plates^25^. Briefly, different concentrations of the indicated peptides were prepared by performing two-fold serial dilutions and were added to an equal volume (100 μL) of bacterial culture in Mueller-Hinton broth in each well of the 96-well plates. The plates were incubated at 37 °C for 18 h. MIC was defined as the concentration at which no microbial growth was observed visually or spectrophotometrically at a wavelength of 600 nm. MBC was defined as the lowest concentration at which no bacterial growth was detected when 200 µL of bacteria and peptide solution, as used in the MIC assay, was plated on agar.

### Haemolysis assay

The haemolytic activity of the peptides was evaluated by determining haemoglobin release from a blood erythrocyte suspension^26^. Briefly, erythrocytes were washed three times with 10 mM phosphate-buffered saline (PBS, pH 7.3) and were centrifuged at 1,300 × *g* and 4 °C for 10 min. Next, 100 μL of erythrocyte suspension diluted with 10 mM PBS (final concentration, approximately 2%) was mixed with 100 μL of each serial two-fold dilution of AMPs (final concentration, 0.5–128 μM). The mixtures were incubated at 37 °C for 1 h. After incubation, the mixtures were centrifuged at 1,300 × *g* for 5 min. The resulting supernatants were transferred into 96-well microtiter plates, and optical density was measured at 540 nm using a Multiskan Spectrum (Thermo Fisher Scientific, USA). Values for 0% and 100% lysis were determined by incubating the erythrocytes with 10 mM PBS and 0.1% (v/v) Triton X-100, respectively.

### Cytotoxicity assay

A549 and HaCaT cells were cultured in DMEM (HyClone) containing 10% FBS (HyClone) under standard conditions in a humidified incubator with 5% CO_2_ at 37 °C. The cytotoxic effect of the AMPs on A549 and HaCaT cells was measured by performing an MTT assay. For this, the cells were seeded (density, 10^4^ cells/well) in a 96-well plate containing 100 μL of culture medium and incubated for 18 h. Next, the cells were incubated with the serial dilutions of the peptides for various time intervals. At the end of the incubation period, cell morphology was determined under an inverted microscope (Olympus IX71) and was analysed using ImagePro Insight software (Olympus). Next, MTT solution (0.5 mg/mL ; Sigma) was added to each well, and the cells were incubated for 3 h at 37 °C. Cell lysis solution (100 μL/well; 20% SDS, 50% *N*,*N-*dimethylformamide) was then added to the wells to dissolve the formazan crystals, and the cells were incubated overnight at 37 °C in a humidified incubator. Absorbance was measured at a wavelength of 570 nm using an ELISA reader (Dynex MRX II; Dynex Technologies, USA).

### Scanning electron microscopy

*A*. *baumannii* strain ATCC 17978 was grown to the log phase in LB broth, harvested by centrifugation, washed twice with PBS, and resuspended in PBS^13^. Approximately 10^9^ cells were incubated with 80 μM LysAB2 P3 at 37 °C for 1 h. Control cells were treated with PBS. The volume was adjusted to 200 μL. The treated cells were fixed with 2.5% (w/v) glutaraldehyde in 0.1 M cacodylate buffer and 1% tannic acid, extensively washed with phosphate buffer, and dehydrated using a graded ethanol series. After critical-point drying and gold coating, the cells were examined under a scanning electron microscope (S-4700; Hitachi, Tokyo, Japan).

### Transmission electron microscopy

Samples containing *A*. *baumannii* strain ATCC 17978 (10^9^ CFU) in Mueller-Hinton medium were incubated with 80 μM LysAB2 P3 for 30 min, followed by centrifugation at 4000 × *g* for 10 min. Cell pellets were fixed with 2.5% (w/v) glutaraldehyde in 0.1 M cacodylate buffer and 1% tannic acid at 4 °C for 1 h and were washed twice with deionized water. A drop containing the bacteria was deposited onto a carbon-coated grid with 2% uranyl acetate, and the grids were examined using a transmission electron microscope (H-7500; Hitachi).

### Bacterial cytoplasmic membrane permeation assay

The ability of the AMPs to permeate the bacterial cytoplasmic membrane was assayed by fluorescein isothiocyanate (FITC) staining, as described by Lai^14^. Briefly, *A*. *baumannii* were grown at 37 °C in LB broth until the absorbance of the culture at 600 nm reached 1. Next, bacterial cells were centrifuged, washed, and resuspended in phosphate buffer. Approximately 10^9^ cells were mixed with 16 μM LysAB2 P3 diluted in phosphate buffer to obtain a final volume of 100 μL (final concentration, 8 μM). A control experiment was performed in the absence of AMPs and in the presence of phosphate buffer. The mixture was incubated in an Eppendorf tube at 37 °C for 1 h. The cell suspension was poured onto glass slides and was maintained at 37 °C for 30 min to allow the adhesion of AMP-treated *A*. *baumannii* cells to the slides. The slides were then washed gently with sodium phosphate buffer and were incubated with 100 μL of FITC solution (6 μg/mL in sodium phosphate buffer; Sigma). After incubation for 30 min at 30 °C, the FITC solution was removed and the slides were rinsed with sodium phosphate buffer. The slides were then examined under a bright-field and fluorescence microscope to assess FITC influx into the bacterial cells.

### Bactericidal kinetics

Time-killing assays were performed as described by Thandar^17^ to evaluate the bactericidal action of AMPs against *A*. *baumannii*. Briefly, bacteria were washed in PBS buffer, resuspended to ~10^6^ CFU/mL, mixed with LysAB2 P3, and incubated for 2 h at room temperature (22–25 °C). At predetermined time points (0, 1, 5, 10, 15, 30, 60, and 120 min), the reactions were serially diluted and plated for enumeration.

### Bactericidal activity in plasma and its components

The bactericidal activities of the AMPs in plasma was assessed by *A*. *baumannii* killing assays in plasma^17^. The experiments and methods associated with human blood received Institutional Review Board (IRB) approval (IRB105-146-A). All participants gave written informed consent. All methods were performed in accordance with the relevant guidelines. Human blood was collected in a heparin tube and centrifuged. The supernatant was filtered and stored at 4 °C overnight. The filtrate was centrifuged and filtered again to remove any debris. The resulting solution was used as 100% plasma. The components of plasma examined for interference were monovalent cations (Na^+^), divalent cations (Ca^2+^ and Mg^2+^), and albumin. Chloride salts of the cations and albumin from human serum (lyophilized powder, ≥97%; Sigma) were prepared in ddH_2_O and sterile filtered. Bactericidal assays, performed as described above, were conducted in 100% plasma and in buffered solutions (50 mM Tris-HCl [pH 7.5]) containing 150 mM NaCl, 1 mM CaCl_2_, 1 mM MgCl_2_, or 50 mg/ml human serum albumin using 64 µM LysAB2 P3. The experiments were conducted at least in triplicate, and the data are shown as the means and standard deviations.

### Determination of endotoxin-releasing and endotoxin-binding activity

To determine the amount of endotoxin released from bacteria after AMP treatments, we performed an LAL assay (Lonza) according to the manufacturer’s instructions. Briefly, *A*. *baumannii* strain 17978 (10^8^ CFU) was incubated with various concentrations of LysAB2 P3 at room temperature for 4 h. Then, the samples were centrifuged (2,000 × g) for 1 min. The supernatant was collected and incubated with LAL reagent. The level of endotoxin was determined by measuring the absorbance at 340 nm on a spectrophotometer (Multiskan Spectrum, Thermo Scientific). To evaluate the endotoxin-binding activity of LysAB2 P3, we performed an endotoxin neutralization assay. A fixed concentration of LPS from *E*. *coli* O55:H9 (2 endotoxin units) was incubated with different concentrations of LysAB2 P3 or colistin at 37 °C for 30 min. After incubation, the LAL reagent was added to each well. The remaining biologically active LPS was quantified according to the procedures described above.

### *In vivo* animal studies

Eight-week-old male BALB/c mice (weight, 20–25 grams) were provided by NLAC Company (Taipei, Taiwan). All experiments involving the mice and all the care and handling of the mice were performed using protocols approved by the Institutional Animal Care and Use Committee of Tzu Chi University (No. 104078). All methods were performed in accordance with the relevant guidelines. *A*. *baumannii* ATCC strain 17978 were grown in LB broth at 37 °C for 18 h, and their concentration was adjusted using physiologic saline. For bacterial load evaluation, the mice were intraperitoneally (i.p.) inoculated with 200 μL/mouse (2 × 10^7^ CFU) of the bacterial solution. At 1 h after the bacterial injection, the mice were i.p. injected with LysAB2 P3 (100 μM/200 μL). Finally, the mice were sacrificed after another 4 h. As a control, PBS (10 mL/kg) was administered at 1 h. Each group included 10 mice. To evaluate the ability of LysAB2 P3 to control the bacterial load and prevent lethal bacteremia, two experimental conditions were tested. The mice were challenged by i.p. injection of *A*. *baumannii* (5 × 10^8^ CFU/mouse, 200 μL/mouse). In the first experiment, at 1 h after the bacterial injection, the mice were treated with 32 μM/mouse (200 μL/mouse) of LysAB2 P3. In the second experiment, at 3 h after the bacterial injection, the mice were treated with 200 μM/mouse (200 μL/mouse) of LysAB2 P3. In both conditions, LysAB2 P3 was given through an intraperitoneal injection, and the mice were exsanguinated after another 4 h. PBS (10 mL/kg) was used as a negative control in both experiments. Each group included 10 mice. Blood samples (100 μL) obtained from the mice were mixed with 38% sodium citrate (10 μL), diluted 100-fold, and plated on LB agar so that the number of bacterial colonies could be counted. The ability of LysAB2 P3 to rescue mice from lethal bacteremia was assayed by the mouse *Acinetobacter* infection sepsis model, as described by Lood^9^. Briefly, thirty male BALB/c mice (6 to 8 weeks of age; NLAC Company) were injected i.p. with *A*. *baumannii* ATCC 17978 (5 × 10^8^ CFU). Three hours after injection, animals were treated i.p. with either LysAB2 P3 (200 μM /200 μL; n = 15) or PBS (n =15), and survival was tracked for 2 weeks.

### Statistical analysis

Data are expressed as the mean ± standard deviation. The statistical significance of all the data was analysed with Student’s t-test. Statistical significance was defined as p <0.05. Statistical analysis of the mouse survival rate evaluations was performed using GraphPad Prism software 6.0 with *p <0.05.

### Ethics statement

All animal experiments were conducted using protocols approved by the Institutional Animal Care and Use Committee of Tzu Chi University (No. 104078). The experiments associated with human blood collection and applications were approved by the Research Ethics Committee of Tzu Chi Hospital (No. IRB105–146A). All the experimental protocols were approved by the ethics committee of Tzu Chi University.
